# Psychometric properties of a self-administered oral health literacy questionnaire for Brazilian adolescents

**DOI:** 10.1590/1980-549720260032

**Published:** 2026-07-27

**Authors:** Helene Soares Moura, Rafael Aiello Bomfim, Paulo Frazao

**Affiliations:** I Universidade de São Paulo, School of Public Health, Department of Policy, Management and Health – São Paulo (SP), Brazil.; II Universidade Federal de Mato Grosso do Sul, School of Dentistry, Department of Social and Preventive Dentistry – Campo Grande (MS), Brazil.

**Keywords:** Health literacy, Oral health, Adolescent, Adolescent health

## Abstract

**Objective:**

To evaluate the psychometric properties of a self-administered oral health literacy (OHL) questionnaire for adolescents, titled the Brazilian Oral Health Literacy-Youngs Questionnaire (BOHL-YQ).

**Methods:**

The study included adolescents aged 15 to 19 years enrolled in public schools in the municipality of Araruna, Paraíba, Brazil. Data on demographic, socioeconomic characteristics, and oral health-related behaviors were collected from 360 participants. The internal consistency of the BOHL-YQ was evaluated. To examine the dimensional structure of the instrument, exploratory factor analysis (EFA) and confirmatory factor analysis (CFA) were conducted. Convergent validity was investigated through associations between BOHL-YQ scores and demographic, socioeconomic, and oral health-related behavioral variables.

**Results:**

A total of 348 questionnaires were analyzed. One item showed inadequate loading in the EFA of the 15-item version. The EFA of the 14-item version demonstrated good internal consistency (Cronbach’s alpha=0.71; McDonald’s omega=0.72), a Kaiser-Meyer-Olkin value of 0.77, and adequate fit for two domains: Cognitive and Comprehension/Decision-Making. The CFA indicated the model explained 84% of the variance. Higher OHL scores were found among female adolescents (p=0.006), urban residents (p<0.001), and those whose mothers had >9 years of schooling (p=0.014), did not receive government benefits (p<0.001), had a dental visit within the last year (p<0.001), and in private dental services (p=0.043).

**Conclusion:**

In this context, the BOHL-YQ demonstrated good internal consistency and convergent validity, indicating that it is a valid and easily applicable instrument for measuring oral health literacy among Brazilian adolescents.

## INTRODUCTION

Health literacy (HL) refers to the skills related to obtaining, understanding, and using health information and services for the promotion and maintenance of health. It is considered a complex and multidimensional construct comprising individual knowledge, skills, and capabilities regarding health and disease, risk and protective factors, and health systems and resources^1^. Oral health literacy (OHL) is a subset of HL that can be defined by the extent to which individuals are able to obtain, process, and understand information regarding oral health, thereby enabling them to make appropriate decisions concerning their oral health^2^. Within the field of health, the use of the term “literacy” has increased in recent years, replacing other terms in Portuguese like “alfabetização” and “letramento”, particularly in studies concerning the management of chronic diseases within the realm of health promotion, as it appears to better capture the multidimensional nature of the theoretical models currently being proposed in the health sciences^3^.

OHL is linked to the degree of individual autonomy and empowerment^2^. Given its associations with socio-demographic characteristics, oral health behaviors, and clinical oral health outcomes^4,5,6,7,8,9^, it may be considered a significant determinant of oral health^4^.

Low levels of OHL have been associated with poorer socioeconomic characteristics^5,6,7^ and poorer oral health conditions across all age groups^8,9,10^. Among Brazilian adolescents, a high level of OHL was a predictor of dental service utilization at age 12^11^ and was associated with greater family cohesion, higher income, and higher maternal educational attainment^6^, as well as a lower number of untreated carious lesions^12^. Among adolescents aged 15 to 19, higher OHL was associated with a lower number of cavitated carious lesions^13^, less tooth loss^14^, and better academic performance^15^; it was also associated with white skin color, a more stable family structure^5^, enrollment in private schools, membership in more affluent economic classes, and parents with higher levels of education^7^. These findings underscore the broad context surrounding this topic; however, knowledge regarding OHL among Brazilian adolescents remains scarce given the diversity of the Brazilian population.

Although a large number of instruments exist^16^, few are specifically designed for assessing OHL in adolescents. For the general Brazilian population, seven OHL instruments have been validated: the Brazilian Rapid Estimate of Adult Literacy in Dentistry (BREALD-30)^17^, the Rapid Estimate of Adult Literacy in Medicine and Dentistry (REALMD-20)^18^, the Oral Health Literacy Assessment-Brazilian (OHLA-B)^19^, the Brazilian version of the Health Literacy in Dentistry scale (HeLD-29 and HeLD-14)^20^, the Brazilian Hong Kong Oral Health Literacy Assessment Task for Pediatric Dentistry (BOHLAT-P)^21^, and the Brazilian Oral Health Literacy-Adults Questionnaire (BOHL-AQ)^22^, of which only two are applicable to the adolescent population.

The BREALD-30, frequently used with Brazilian adolescents^5,6,7,11,12,13^, requires an interviewer. The HeLD-14 is self-administered and measures functional and communicative OHL^15^. Since the choice of questionnaire depends on the specific context and available resources, it is important to expand the range of tools available for measuring OHL among adolescents, as well as the skills encompassed by this construct.

The objective of this study was to evaluate the psychometric properties of a self-administered oral health literacy questionnaire for adolescents aged 15 to 19, titled the Brazilian Oral Health Literacy-Youngs Questionnaire (BOHL-YQ).

## METHODS

A cross-sectional study was conducted to assess the psychometric properties, specifically structural and convergent validity, of a measurement instrument designed for population-based research on OHL among Brazilian adolescents aged 15 to 19.

The study adhered to the ethical principles of the Declaration of Helsinki and Brazilian regulatory standards (CAAE: 73709623.1.0000.5421). Participants aged 18 or older, and also the guardians of those under 18, received prior information regarding the study; an informed consent form was subsequently signed.

The article followed expert recommendations for reporting studies on health measurement instruments^23^.

### Instrument

The BOHL-YQ questionnaire was initially evaluated with 15 items distributed across 12 questions. This instrument was based on the BOHL-AQ, which was translated, culturally adapted, and validated by Almeida et al.^22^. The source of this instrument, adapted for Brazilian Portuguese, is the Oral Health Literacy-Adults Questionnaire (OHL-AQ), developed by Sistani et al.^24^ in Iran.

The BOHL-AQ^22^, comprising 17 items distributed across 14 questions, was administered to a broad age range (18 to 71 years) of employees at a private university located in southeastern Brazil. The validation of the Brazilian version included an intercultural adaptation process, which involved translation into Brazilian Portuguese; an assessment of conceptual equivalence by experts; approval of the version by the original instrument’s author; and a pilot test conducted with 20 individuals. Although originally developed to assess four functional literacy skills, its psychometric properties indicated a unidimensional structure^22^.

To render the questionnaire entirely self-administered, two questions pertaining to the listening domain, which required administration by an interviewer, were excluded from the original questionnaire. Consequently, the participant reads the questions and selects the responses without assistance from the administrator, whose role is limited to explaining how to complete the questionnaire, checking for missing items, and providing the respondent with the opportunity to finalize their responses. Thus, the questionnaire utilized contained 12 questions and a score ranging from 0 to 15, given that Question 2 is subdivided into 3 scorable items and Question 3 into 2 scorable items.

To verify the appropriateness of the language, this 15-item version was submitted to a group of students sharing the same socioeconomic profile and age range, but who were not part of the study population. To this end, a focus group interview was used, an exploratory methodological procedure utilizing a qualitative and contextual approach, as recommended for this purpose^25,26^. After obtaining consent, the adolescents were invited to complete the questionnaire and note down any questions they might have. Subsequently, during a group discussion session involving the adolescents and a moderator, each item of the instrument was read aloud, and the difficulties encountered by the adolescents were brought to light. For certain questions, the only difficulty identified related to the specific knowledge required to answer the, an outcome that was anticipated.

Only one question presented a text comprehension challenge for the majority of the adolescents: “In your opinion, what is the meaning of the phrase ‘I exempt my dentist from unintentional complications of the treatment’”? The term “exempt” was unfamiliar to the adolescents’ vocabulary; among the alternatives discussed by the group, the decision was made to replace it with the term “absolve”, to preserve the content and objective of the question.

### Study population

The study participants were adolescents aged 15 to 19 who were actively enrolled in the morning and afternoon shifts of the two public high schools in the city of Araruna, PB, and who had previously submitted the necessary authorizations.

### Administration of the instrument

The BOHL-YQ questionnaire was administered to adolescents in a classroom setting in May 2024. Data regarding demographic and socioeconomic characteristics, as well as behaviors related to oral health, were also collected using questions drawn from the 2019 National Survey of School Health (PeNSE) by the Brazilian Institute of Geography and Statistics (IBGE), and the 2023 National Oral Health Survey (SB Brasil) by the Ministry of Health.

Of the 614 students with active enrollment, 360 adolescents returned the required authorization forms and completed the questionnaires, resulting in a participation rate of 58.6%.

### Statistical analysis

The internal consistency of the questionnaire was measured using Cronbach’s alpha^27^ and McDonald’s omega^28^. The correlation matrix between the items was determined, and the null hypothesis of an absence of correlation between the variables was analyzed (Bartlett’s test of sphericity). If there was an indication of the existence of correlation, exploratory factor analysis (EFA) was justified; in this analysis, Kaiser-Meyer-Olkin (KMO) values ≥0.5 were considered adequate^29^. The EFA was performed using principal components analysis to assess dimensionality. Eigenvalues greater than 1 were retained as principal factors; subsequently as there was no empirical evidence of a relationship between the factors, rotation followed the orthogonal Varimax method, executed to assign weights to each item within each retained factor, where values over 0.4 were considered to define each factor^30^. Stata^®^ software v.14.2 was used.

The structural validity and dimensionality of the instrument were investigated using confirmatory factor analysis (CFA), using the robust maximum likelihood method because of the ordinal nature of the instrument’s data. To assess the goodness-of-fit of the models, the following criteria were considered: comparative fit index (CFI) >0.90, Tucker-Lewis index (TLI) >0.90, root mean square error of approximation (RMSEA) <0.06, standardized root mean square residual (SRMR) <0.05 ^29,30^, and Cronbach’s alpha (α) ≥0.731. Reliability was evaluated using McDonald’s omega coefficient (Ω) ≥ 0.70^28^.

Two models were run to evaluate goodness-of-fit. The EFA ofthe 15-item questionnaire indicated the presence of one item with an unsatisfactory factor loading (<0.4)^30^. This item was excluded, and a new EFA was performed with the remaining 14 items, resulting in a more consistent structure. Subsequently, CFA was applied based on this 14-item model to verify the instrument’s goodness of fit.

Descriptive statistics were performed for demographic and socioeconomic characteristics, as well as for behaviors related to oral health; data distribution normality was assessed using the Shapiro-Wilk test. OHL levels were defined similarly to the criteria suggested by the reference studies cited by Sistani et al.^24^, in which the lowest level is represented by the range of values that bisects the scale, while the remaining levels are distributed equally across the remaining values. Thus, scores between 0 and 6 were classified as low/inadequate; scores from 7 to 10 as intermediate; and scores from 11 to 14 as adequate/high.

Convergent validity was examined by comparing differences in total mean scores and mean scores per domain across socioeconomic and demographic variables, and also time elapsed since the last dental visit and type of dental service used, with the help of the Mann-Whitney U and Kruskal-Wallis tests via the statistical software Jamovi^®^ (version 2.3.28).

Sample size calculation remains a challenge in structural equation modeling. Traditional approaches suggest samples comprising a minimum of 200 individuals, or ideally meeting a ratio of 20 observations per parameter. More recent methods employ various simulation techniques^32^. Given that the sample size in this study exceeded 280 observations, the parameters of both approaches were considered satisfied, thereby ensuring 80% statistical power at an alpha level of 5%.

#### Data Availability Statement:

The whole dataset supporting the results of this study is available from the corresponding author H.S. Moura upon request. The dataset is not publicly available to protect the identity and privacy of the participants and to comply with ethical requirements.

## RESULTS

Twelve observations with incomplete data were excluded, and 348 valid questionnaires were analyzed.

The 15-item instrument demonstrated good internal consistency (Cronbach’s alpha =0.71) and variable adequacy for EFA: Bartlett’s test of sphericity (p=0.000) and KMO =0.77. The EFA indicated five main factors (eigenvalues >1). After rotation using the orthogonal Varimax method, the five factors explained 51.7% of the variability in the findings. However, Item 11, corresponding to Question 8, “What is the best decision if minor bleeding occurs after brushing or flossing”? exhibited a factor loading of <0.4^30^, indicating that it did not assess any of the retained factors; it was, therefore, removed from the instrument. The removal of this question resulted in a final instrument comprising 14 scorable items distributed across 11 questions, with a score ranging from 0 to 14 points. Consequently, a second EFA was performed using the 14 items. Good internal consistency (α=0.71) and adequacy for factor analysis (KMO=0.77) were maintained.

Table 1 presents the factor loadings of the BOHL-YQ questionnaire items following exploratory factor analysis (EFA) with orthogonal rotation, distributed across five factors. Based on the nature of the questions, seven questionnaire items associated with the Cognitive domain (items 1, 2, 3, 4, 5, 6, and 12) exhibited higher factor loadings on Factors 2, 4, and 5, whereas Factors 1 and 3 clustered items focused on Comprehension and Decision-making (items 7, 8, 9, 10, 11, 13, and 14), suggesting the testing of a two-dimensional structure. In cases where loadings exceeded 0.4 on more than one factor, the decision was made to select the highest loading and assign the item to the domain where it fit best. Most items exhibited low uniqueness (<0.50), indicating good communality between the items and the extracted factors.

**Table 1:** Factor loadings distributed by domain according to the items of the Brazilian Oral Health Literacy-Youngs Questionnaire (BOHL-YQ), following orthogonal rotation. (n=348).

Domain	Loadings						
	Variable	Factor 1	Factor 2	Factor 3	Factor 4	Factor 5	Unit
Cognitive	Item 1	0.11	-0.04	-0.05	0.11	**0.76**	0.39
	Item 2	0.43	**0.54**	0.00	0.11	0.11	0.50
	Item 3	0.07	**0.71**	0.00	0.16	-0.19	0.43
	Item 4	0.42	**0.58**	0.05	-0.02	-0.08	0.48
	Item 5	0.14	0.03	-0.06	**0.74**	-0.05	0.42
	Item 6	-0.02	0.08	0.09	**0.78**	0.18	0.35
	Item 12	-0.11	**0.62**	0.21	-0.01	0.39	0.41
Comprehension and decision-making	Item 7	**0.52**	0.17	0.10	0.35	-0.22	0.51
	Item 8	0.09	0.08	**0.57**	0.15	0.20	0.57
	Item 9	0.18	-0.06	**0.74**	0.05	-0.02	0.42
	Item 10	**0.64**	0.13	0.29	0.10	-0.16	0.45
	Item 11	0.07	0.26	**0.62**	-0.11	-0.12	0.51
	Item 13	**0.77**	0.01	0.03	0.01	0.17	0.37
	Item 14	**0.58**	0.15	0.18	0.01	0.35	0.49

Note: In bold are values over 0.4 that explain each factor.

Subsequently, a confirmatory analysis for a two-dimensional structure was conducted using structural equation modeling. The obtained fit indices were: comparative fit index (CFI)=0.88; non-normed fit index (NNFI)=0.86; root mean square error of approximation (RMSEA)=0.05 (90%CI 0.03–0.06); standardized root mean residual (SRMR)=0.05; χ^2^/degrees of freedom p=0.000; and coefficient of determination (CD)=0.84, meaning the model explained 84% of the variability in the findings. Ω=0.72 indicated good overall internal consistency of the scale, suggesting that the items adequately measure the same latent construct.

**Figure 1:**
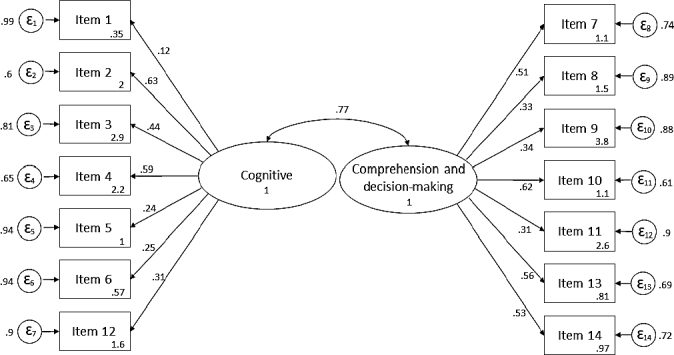
Standardized coefficients (values in the row) considering 95%CI, standardized factor loadings (values inside the rectangle), and estimated residual variance for the 14 items of the Brazilian Oral Health Literacy-Youngs Questionnaire (BOHL-YQ), according to the structural equation model.

**Figure 2:**
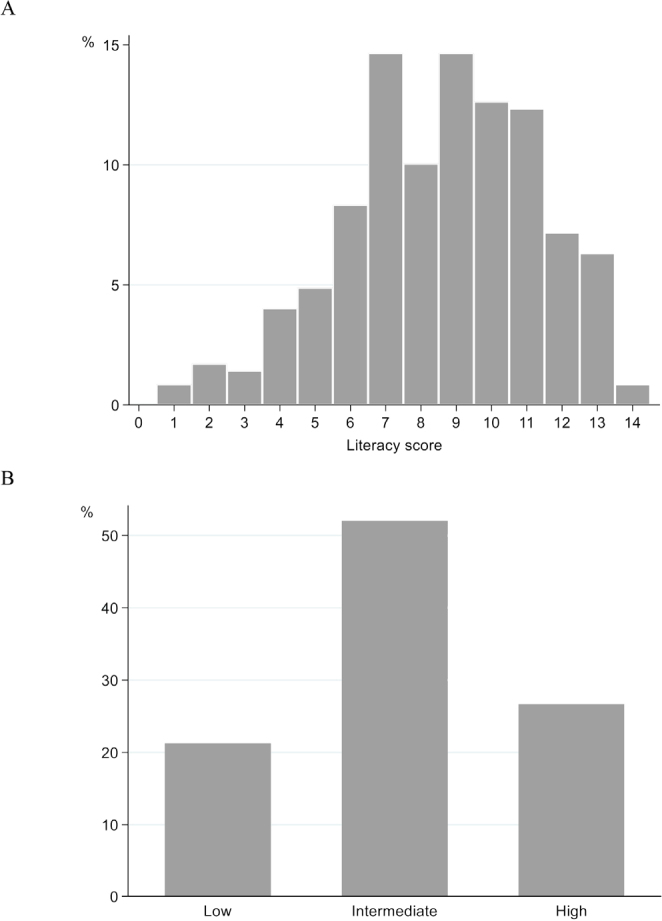
Distribution of the study population: (A) according to oral health literacy score and (B) according to level of low/inadequate OHL (scores of 0 to 6), intermediate (scores of 7 to 10), and adequate/high (scores of 11 to 14).

Figure 1 presents the adjusted coefficients for the 14 items of the BOHL-YQ questionnaire (Supplemental Table 1), based on the structural equation model. Regarding the sample distribution according to OHL level, illustrated in Figure 2, 21.3% of the adolescents exhibited a low/inadequate level, 52% an intermediate level, and 26.7% a high/adequate level.

Table 2 presents the mean scores for each domain and the total score, categorized by demographic and socioeconomic characteristics, as well as behaviors related to oral health. The following was found for the total sample: 62.6% were girls; 63.8% self-identified as having brown or black skin color; 93.1% reported owning a cell phone; 53.2% resided in an urban area; 39.4% lived in a household with up to three residents (including the adolescent themselves); 61.5% stated that their family received some form of government benefit; 35.3% reported that their mother had either not attended school or had not completed elementary education; 54.6% reported having visited a dentist within the past year; and 60.9% reported having used public services for their dental visit.

**Table 2:** Mean score and standard deviation overall and for each domain of the Brazilian Oral Health Literacy-Youngs Questionnaire (BOHL-YQ), according to socioeconomic and demographic characteristics and oral health-related behaviors of the study population.

Variables	n	%	Cognitive	Comprehension and decision-making	Total score
			Mean (SD)	Mean (SD)	Mean (SD)
**Sex**					
Female	218	62.6	4.20 (1.39)	4.66 (1.66)^**^	8.86 (2.63)^**^
Male	130	37.4	3.97 (1.49)	4.12 (1.87)	8.08 (2.84)
**Skin color self-declared**					
White	108	31.0	4.26 (1.43)	4.67 (1.84)	8.93 (2.78)
Non-white	240	69.0	4.05 (1.43)	4.36 (1.71)	8.41 (2.70)
**Owns cell phone?**					
Yes	324	93.1	4.11 (1.44)	4.50 (1.75)	8.61 (2.72)
No	24	6.9	4.13 (1.39)	3.88 (1.80)	8.00 (2.84)
**Area of residence**					
Urban	185	53.2	4.31 (1.33)^**^	4.75 (1.76)^***^	9.06 (2.56)^***^
Rural	163	46.8	3.88 (1.51)	4.13 (1.70)	8.01 (2.82)
**Number of household members**					
Up to 3	137	39.4	4.26 (1.49)	4.53 (1.75)	8.80 (2.81)
4	109	31.3	4.07 (1.43)	4.61 (1.76)	8.69 (2.76)
5 or more	102	29.3	3.95 (1.34)	4.19 (1.74)	8.14 (2.58)
**Mother’s education level**					
≤9 years	196	57.5	4.03 (1.44)	4.38 (1.80)	8.40 (2.83)
>9 years	122	35.8	4.24 (1.36)	4.72 (1.64)^**^	8.96 (2.48)^**^
Not known	23	6.7	3.74 (1.57)	3.57 (1.56)	7.30 (2.62)
**Government benefit**					
Yes	214	61.5	3.97 (1.49)	4.36 (1.75)	8.32 (2.79)
No	93	26.7	4.56 (1.28)^***^	4.91 (1.63)^**^	9.47 (2.40)^***^
Not known	41	11.8	3.85 (1.26)	3.95 (1.90)	7.80 (2.71)
**Last dentist visi**t					
Within last year	190	54.6	4.27 (1.41)	4.80 (1.71)^***^	9.07 (2.68)^***^
More than 1 year ago	116	33.3	3.98 (1.46)	4.03 (1.76)	8.02 (2.73)
Not known	42	12.1	3.71 (1.35)	4.00 (1.63)	7.71 (2.44)
**Location of last visit**					
Public service	212	60.9	4.09 (1.44)	4.42 (1.74)	8.51 (2.73)
Private service	102	29.3	4.27 (1.37)	4.72 (1.78)	8.99 (2.60)^*^
Not known	34	9.8	3.74 (1.54)	3.91 (1.71)	7.65 (2.92)^*^
**Total**	**348**	**100**			

SD: standard deviation;

*p<0.05;

**p≤0.01;

***p≤0.001.

Regarding convergent validity, statistically higher mean OHL scores were observed among the following: female adolescents (p=0.006); those residing in urban areas (p<0.001); those whose mothers had more than nine years of formal education (p=0.014); those whose families did not receive government benefits (p<0.001); those who had their last dental visit within the past year (p<0.001); and those who used private services for their dental visit (p=0.043). Mean scores were more favorable for the Cognitive domain among the following: residents of urban areas (p=0.003); those not participating in government benefit programs (p<0.001); and those whose last dental visit occurred within the past year (p=0.023). Conversely, for the Comprehension and Decision-making domain, higher mean scores were identified among females (p=0.005), those with mothers who had more than 9 years of schooling (p=0.011), those residing in urban areas (p=0.001), those not receiving government benefits (p=0.013), and those whose last dental visit occurred within the past year (p<0.001).

## DISCUSSION

The BOHL-YQ instrument, tested to measure OHL among Brazilian adolescents aged 15 to 19, demonstrated adequate internal consistency, as well as good structural and convergent validity. These results were similar to those of the original Iranian version (α=0.72)^24^ and the Brazilian version (α=0.73)^22^, both of which were designed for adults.

The BREALD-30, an instrument frequently used with Brazilian adolescents, demonstrated higher internal consistency than the BOHL-YQ (α=0.83), yet yielded similar convergent validity results: higher levels of oral health literacy were observed among adolescents from more favorable economic backgrounds and those with parents possessing higher levels of education^7^.

The mean OHL score was higher among female adolescents, a finding consistent with that reported by Lima et al.^7^ in a study involving 12-year-old adolescents. The OHL scores were higher with higher maternal education levels and better family socioeconomic status, represented in this study by the absence of enrollment in any government social assistance program, was also observed by Lopes et al.^5^ in a study involving adolescents within the same age range.

Adolescents who reported having had their last dental visit within the past year exhibited higher mean OHL scores, both in terms of the total score and across individual domains. Higher OHL scores appear to be associated with a greater awareness of oral health care during adolescence and, consequently, with more positive health-related behaviors.

Although the distribution of characteristics within the study population allowed for the detection of differences between OHL scores and various demographic, socioeconomic, and oral health-related behavioral variables, and given that instrument validation entails the synthesis of evidence from multiple sources, it is suggested that this instrument be further tested within diverse Brazilian socioeconomic and cultural contexts. Of the total adolescent sample, 21.3% demonstrated a low or inadequate level of OHL. As the categorization of OHL scores into three distinct levels was based on suggestions found in the previous literature^33,34^, it is recommended that this proposed classification scheme be subjected to further scrutiny in future studies.

Although not all parameters for the psychometric analysis of health-related outcome measures were investigated^24^, the BOHL-YQ yielded satisfactory results across all proposed analyses. The EFA indicated a five-factor structure, with a coherent distribution across the proposed theoretical domains: Cognitive and Comprehension/Decision-making. The choice of orthogonal rotation represented an approach compatible with the exploratory nature of the analysis, favoring a more parsimonious solution and a more direct interpretation of the factor groupings. However, given that this is a multidimensional psychosocial construct, the possibility of correlation between the identified domains cannot be ruled out; future studies could explore oblique rotations as a complementary strategy to assess the stability and interpretability of the resulting structure.

In the CFA, the RMSEA and SRMR indices fell within acceptable limits, while the NNFI (0.86) and CFI (0.88) indices bordered on the reference parameters described in the methodology. Since the evaluation of results should employ a set of measures rather than relying on a single reference value^35^, the model fit was deemed satisfactory and theoretically consistent with the proposed structure. Consequently, the decision was made to retain the model, while acknowledging this limitation and emphasizing that fit indices constitute only one aspect of model evaluation^35^.

Although the authors stated that the original questionnaire, which served as the basis for the instrument validated in this study, assessed four dimensions related to conceptual components of health literacy (reading comprehension, numeracy, listening, and decision-making)^22^, only a unidimensional CFA of that instrument had been reported.

The satisfactory internal consistency values and the CFA results demonstrate that the final structure of the instrument exhibits sound psychometric properties, maintaining theoretical coherence while enhancing the interpretability of the findings. The consolidation into two broader domains (Cognitive and Comprehension/Decision-making) resulted in a more parsimonious structure. The assessment of these two domains of the OHL construct using a single instrument enables a more detailed analysis and can identify strengths and weaknesses for each of them, thereby aiding in the design and monitoring of potential practical OHL interventions with adolescents.

In the present study, two items exhibited adequate factor loadings on more than one factor, which may be related to the characteristics of the study population. Although the general structure indicates a satisfactory theoretical grouping, the overlap of an item across multiple factors suggests the need for item refinement through future revisions of the scale, specifically regarding the wording of certain items, and the potential for testing using oblique rotation, which can capture correlations between factors. However, both EFA and CFA were conducted on the same sample, which limits the robustness of the evidence regarding structural validity. While this strategy can be useful in initial psychometric evaluations, the absence of sample splitting, cross-validation, or testing on an independent sample may yield more optimistic fit estimates. Thus, the findings should be interpreted as preliminary evidence of the BOHL-YQ’s factorial structure — evidence that requires further elaboration in future investigations involving replication across other adolescent populations.

The failure to assess convergent validity by comparing the BOHL-YQ against another OHL instrument previously validated for the same population may also be considered a limitation. Nevertheless, the results suggest that the instrument possesses preliminary psychometric adequacy for application in Brazilian adolescent populations, thereby justifying its confirmatory validation in subsequent studies.

Compared to the BREALD-30, which requires an interviewer and measures term recognition^17^, the BOHL-YQ may offer advantages by taking into account the complexity and multidimensionality of literacy. Beyond merely assessing an individual’s prior knowledge regarding oral health, the BOHL-YQ incorporates stages involving interpretation, calculation, and decision-making in the face of basic oral health-related problems; as such, it possesses the capacity to evaluate a spectrum ranging from cognitive aspects to comprehension and decision-making skills. Furthermore, this self-administered version of the questionnaire enhances practicality by facilitating the testing and utilization of this instrument across a variety of contexts.

The HeLD-14 measures oral health-related quality of life (OHRQoL) using questions based on the individual’s self-perception regarding their level of difficulty in performing activities related to oral health^20^. Although the BOHL-YQ measures only two dimensions, this may represent an advantage for interpreting the global OHRQoL score compared to the HeLD-14, whose score is derived from seven domains some of which are quite distinct^20^.

In the investigated context, it was concluded that the BOHL-YQ demonstrated good internal consistency and convergent validity, proving to be a valid and easily administered instrument for measuring OHRQoL among Brazilian adolescents.
