# Supplementary material for: Psychometric properties of a self-administered oral health literacy questionnaire for Brazilian adolescents
Source: Rev Bras Epidemiol. 2026 Jul 27;29:e260032. doi: 10.1590/1980-549720260032 (PMC13440506; doi:10.1590/1980-549720260032)
Supplement: Supplementary file 1 [file 1980-5497-rbepid-29-e260032-supp1.pdf]

Tabela Suplementar 1. Coeficientes padronizadas e intervalos de confiança (IC95%) do modelo de equações estruturais (BOHL-YQ).

Item	Fator	CP (λ)	IC95%
Item 1	Cognitivo	0,12	-0,01; 0,24
Item 2		0,63	0,54; 0,73
Item 3		0,44	0,33; 0,55
Item 4		0,59	0,49; 0,69
Item 5		0,24	0,12; 0,36
Item 6		0,25	0,13; 0,37
Item 12		0,31	0,19; 0,43
Item 7	Compreensão e Tomada de decisão	0,51	0,41; 0,61
Item 8		0,33	0,21; 0,44
Item 9		0,34	0,23; 0,45
Item 10		0,62	0,53; 0,71
Item 11		0,31	0,20; 0,43
Item 13		0,56	0,46; 0,65
Item 14		0,53	0,43; 0,63

Nota: CP = Coeficiente Padronizado; IC = Intervalo de Confiança.
