# Supplementary material for: Psychometric properties of a self-administered oral health literacy questionnaire for Brazilian adolescents
Source: Rev Bras Epidemiol. 2026 Jul 27;29:e260032. doi: 10.1590/1980-549720260032 (PMC13440506; doi:10.1590/1980-549720260032)
Supplement: Translation

# Translation: Propriedades psicométricas de um questionário autoaplicável de literacia em saúde bucal para adolescentes brasileiros

**DOI:** 10.1590/1980-549720260032.2

**Authors:** Helene Soares Moura, Rafael Aiello Bomfim, Paulo Frazao

**Affiliations:** I Universidade de São Paulo, Faculdade de Saúde Pública, Departamento de Política, Gestão e Saúde – São Paulo (SP), Brasil.; II Universidade Federal de Mato Grosso do Sul, Faculdade de Odontologia, Departamento de Odontologia Social e Preventiva – Campo Grande (MS), Brasil.

**Keywords:** Letramento em saúde, Saúde bucal, Adolescente, Saúde do adolescente

## Abstract

**Objetivo:**

Avaliar as propriedades psicométricas de um questionário de Literacia em Saúde Bucal (LSB) autoaplicável para adolescentes denominado *Brazilian Oral Health Literacy – Youngs Questionnaire* (BOHL-YQ).

**Métodos:**

Participaram adolescentes de 15 a 19 anos de escolas públicas da cidade de Araruna, Paraíba. Foram coletados dados sobre características demográficas, socioeconômicas e comportamentos relacionados à saúde bucal de 360 participantes. Avaliou-se a consistência interna do BOHL-YQ. Para examinar a estrutura dimensional do instrumento, realizou-se Análise Fatorial Exploratória (AFE) e Análise Fatorial Confirmatória (AFC). A validade convergente foi investigada por associação entre os escores do BOHL-YQ e características demográficas, socioeconômicas e de comportamentos relacionadas à saúde bucal.

**Resultados:**

Do total de questionários respondidos, 348 foram analisados. Um item apresentou valor insatisfatório na AFE do questionário com 15 itens. A AFE da versão com 14 itens demonstrou boa consistência interna (alfa de Cronbach = 0,71; ômega de McDonald = 0,72), Kaiser-Meyer-Olkin = 0,77 e bom ajuste para dois domínios do instrumento: Cognitivo e Compreensão/Tomada de decisão. Segundo a AFC, o modelo explicou 84% da variabilidade. Valores significativamente mais elevados de LSB foram observados entre adolescentes do sexo feminino (p=0,006), residentes da área urbana (p<0,001), cujas mães têm mais de 9 anos de estudo (p=0,014), que não recebem benefício do governo (p<0,001) e realizaram última consulta odontológica há até um ano (p<0,001) em serviço privado (p=0,043).

**Conclusão:**

Neste contexto, o BOHL-YQ apresentou boa consistência interna e validade convergente, demonstrando ser um instrumento válido e de fácil aplicação para mensurar LSB entre adolescentes brasileiros.

## INTRODUÇÃO

A Literacia em Saúde (LS) refere-se às habilidades relacionadas à obtenção, compreensão e ao uso das informações e dos serviços de saúde para promoção e manutenção da saúde. É considerado um construto complexo e multidimensional de conhecimentos, habilidades e capacidades individuais sobre saúde-doença, fatores de risco/proteção, e sobre os sistemas e recursos de saúde^1^. A Literacia em Saúde Bucal (LSB) é um subconjunto da LS que pode ser definido pela extensão em que os indivíduos são capazes de obter, processar e compreender informações sobre saúde bucal, permitindo-lhes tomar decisões adequadas em relação à saúde bucal^2^. Na Saúde, o uso do termo “literacia” tem crescido nos últimos anos, em substituição ao termo “letramento”, principalmente associado a estudos sobre o controle de condições crônicas de doenças na área da promoção da saúde, pois parece melhor expressar o caráter multidimensional dos modelos teóricos que vêm sendo propostos na Saúde^3^.

A LSB tem relação com o grau de autonomia e empoderamento dos indivíduos^2^. Por suas relações com características sociodemográficas, comportamentos de saúde bucal e desfechos clínicos bucais^4,5,6,7,8,9^, pode ser considerada um importante determinante de saúde bucal^4^.

Níveis baixos de LSB têm sido associados a piores características socioeconômicas^5,6,7^ e piores condições de saúde bucal em todas as faixas etárias^8,9,10^. Entre adolescentes brasileiros, nível alto foi preditor do uso de serviços odontológicos aos 12 anos^11^ e estava associado a melhor coesão familiar, maior renda e nível de escolaridade materna^6^, e a menor quantidade de lesões de cárie não tratadas^12^. Entre adolescentes de 15 a 19 anos, maior LSB foi relacionada com menor número de lesões cariosas cavitadas^13^, menor perda dentária^14^ e melhor desempenho escolar^15^; cor da pele branca, melhor estrutura familiar^5^, matrícula em escola privada, classes econômicas mais favorecidas e pais com maior escolaridade^7^. Esses achados reforçam o amplo contexto que envolve essa temática, mas o conhecimento sobre LSB dos adolescentes brasileiros ainda é escasso diante da diversidade da população brasileira.

Embora exista grande quantidade de instrumentos^16^, poucos são voltados à LSB de adolescentes. Para a população brasileira em geral, sete instrumentos de LSB foram validados: *Brazilian Rapid Estimate of Adult Literacy in Dentistry* (BREALD-30)^17^, *Rapid Estimate Adult Literacy in Medicine and Dentistry* (REALMD-20)^18^, *Oral Health Literacy Assessment-Brazilian* (OHLA-B)^19^, versão brasileira do *Health Literacy Dental scale* (HeLD-29 e HeLD-14)^20^, *Brazilian Hong Kong Oral Health Literacy Assessment Task for Pediatric Dentistry* (BOHLAT-P)^21^ e *Brazilian Oral Health Literacy-Adults Questionnaire* (BOHL-AQ)^22^ – dos quais apenas dois aplicam-se ao público adolescente.

O BREALD-30, frequentemente utilizado em adolescentes brasileiros^5,6,7,11,12,13^, necessita de um entrevistador. O HeLD-14 é utilizado de forma autoaplicada e mede a LSB funcional e comunicativa^15^. Como a escolha do questionário depende do contexto e dos recursos disponíveis, é importante a ampliação de ferramentas para medir a LSB entre adolescentes e as habilidades que envolvem esse construto.

O objetivo deste estudo foi avaliar as propriedades psicométricas de um questionário de literacia em saúde bucal autoaplicável para adolescentes de 15 a 19 anos intitulado *Brazilian Oral Health Literacy – Youngs Questionnaire* (BOHL-YQ).

## MÉTODOS

Realizou-se um estudo transversal para medir as propriedades psicométricas relativas à validade estrutural e à validade convergente de um instrumento de aferição voltado para pesquisas populacionais sobre LSB entre adolescentes brasileiros de 15 a 19 anos.

O estudo seguiu os preceitos éticos da Declaração de Helsinque e as normas brasileiras (CAAE: 73709623 .1.0000.5421). Os participantes maiores de 18 anos ou responsáveis pelos menores de 18 anos foram previamente esclarecidos, e os Termos de Consentimento e de Assentimento Livre e Esclarecido foram assinados.

O artigo seguiu recomendações de especialistas para comunicação de estudos sobre instrumentos de medição em saúde^23^.

### Instrumento

O questionário BOHL-YQ foi avaliado inicialmente com 15 itens distribuídos em 12 questões. Este instrumento teve por base o BOHL-AQ, traduzido, adaptado culturalmente e validado por Almeida et al.^22^ A origem deste instrumento adaptado para o português brasileiro é o *Oral Health Literacy* – *Adults Questionnaire* (OHL-AQ), desenvolvido por Sistani et al.^24^ no Irã.

O BOHL-AQ^22^, composto de 17 itens distribuídos em 14 questões, foi aplicado a uma faixa etária ampla, de 18 a 71 anos, de funcionários pertencentes a uma universidade privada localizada no Sudeste do Brasil. A validação da versão brasileira incluiu uma adaptação intercultural, que envolveu tradução para o português brasileiro; avaliação da equivalência conceitual com especialistas; aprovação da versão pelo autor do instrumento original; e realização de um pré-teste com 20 indivíduos. Embora desenvolvido originalmente para avaliar quatro habilidades da literacia funcional, suas propriedades psicométricas descreveram uma estrutura unidimensional^22^.

Para tornar o questionário totalmente autoaplicável, foram excluídas do questionário original duas questões relativas ao domínio da escuta, que exigiam sua aplicação por um entrevistador. Desta forma, o participante realiza a leitura das perguntas e escolhe as respostas sem ajuda do aplicador, que pode apenas explicar como preencher o questionário, examinar se há itens faltantes e dar a oportunidade de o respondente finalizar o preenchimento. Assim, o questionário utilizado continha 12 questões e escore variando de 0 a 15, uma vez que a questão 2 é subdividida em 3 itens pontuáveis e a questão 3, em 2 itens pontuáveis.

Para fins de verificação da adequação da linguagem, esta versão de 15 itens foi submetida a um grupo de estudantes de mesmo perfil socioeconômico e faixa etária, mas que não compuseram a população do estudo. Para tal, foi utilizada uma entrevista tipo grupo focal, procedimento metodológico exploratório, de abordagem qualitativa e contextual, recomendado para esta finalidade^25,26^. Após a obtenção do consentimento, os adolescentes foram convidados a responder ao questionário e registrar possíveis dúvidas. Em seguida, em uma roda de conversa dos adolescentes com um mediador, foi realizada a leitura de cada questão do instrumento e expostas as dificuldades dos adolescentes. Para algumas questões foi identificada apenas a dificuldade sobre o conhecimento exigido para respondê-las, o que era esperado.

Apenas uma questão desencadeou dificuldade de compreensão de texto para a maioria dos adolescentes: “Na sua opinião, qual é o significado da frase ‘Eu isento o meu dentista de complicações não intencionais do tratamento’?”. O termo “isento” não era familiar ao vocabulário dos adolescentes; entre as alternativas discutidas em grupo, a opção foi substituir pelo termo “inocento”, a fim de preservar o conteúdo e objetivo da questão.

### População do estudo

Participaram do estudo adolescentes de 15 a 19 anos com matrícula ativa nos turnos matutino e vespertino das duas escolas públicas de ensino médio da cidade de Araruna/PB que trouxeram previamente as devidas autorizações.

### Aplicação do instrumento

O questionário BOHL-YQ foi aplicado em maio de 2024 em ambiente de sala de aula aos adolescentes. Foram também coletados dados sobre as características demográficas, socioeconômicas e de comportamentos relacionados à saúde bucal por meio de questões extraídas da Pesquisa Nacional de Saúde do Escolar (PeNSE) 2019, do Instituto Brasileiro de Geografia e Estatística (IBGE); e da Pesquisa Nacional de Saúde Bucal (SB Brasil) 2023, do Ministério da Saúde.

Dos 614 estudantes com matrícula ativa, 360 adolescentes trouxeram a devida autorização e responderam aos questionários, obtendo-se 58,6% de aderência.

### Análise estatística

A consistência interna do questionário foi medida pelo alfa de Cronbach^27^ e pelo ômega de McDonald^28^. Foi determinada a matriz de correlação entre as questões e analisada a hipótese nula de ausência de correlação entre as variáveis (teste de esfericidade de Bartlett). Havendo o indicativo da existência de correlação, justificase a Análise Fatorial Exploratória (AFE), na qual valores de Kaiser-Meyer-Olkin (KMO) ≥ 0,5 foram considerados adequados^29^. A AFE foi realizada usando análise de componentes principais para avaliar a dimensionalidade. Autovalores maiores do que 1 foram retidos como fatores principais; em seguida, por não haver evidência empírica de relação entre os fatores, a rotação seguiu o método ortogonal Varimax, executada para a atribuição do peso para cada item de cada fator retido, onde valores acima de 0,4 explicariam cada fator^30^. Utilizou-se o software *Stata* ^®^ v.14.2.

A validade estrutural e a dimensionalidade do instrumento foram investigadas por Análise Fatorial Confirmatória (AFC), usando o método de *Robust Maximum Likelihood*, em razão dos dados ordinais do instrumento. Para acessar a qualidade de ajuste dos modelos, foram considerados *Comparative Fit Index* (CFI) > 0,90, Tucker-Lewis Index (TLI) > 0,90, *Root Mean Square Error of Approximation* (RMSEA) < 0,06, *Standardized Root Mean Square Residual* (SRMR) < 0,05^29,30^ e alfa de Cronbach (α) ≥ 0,7^31^. A confiabilidade foi avaliada utilizando o coeficiente ômega de McDonald (Ω) ≥ 0,70^28^.

Foram rodados dois modelos para avaliar a qualidade de ajuste. A AFE do questionário com os 15 itens indicou a presença de um item com carga fatorial insatisfatória (<0,4)^30^. Esse item foi excluído, e uma nova AFE foi realizada com 14 itens, resultando em uma estrutura mais consistente. Posteriormente, a AFC foi aplicada com base nesse modelo de 14 itens, a fim de verificar a qualidade de ajuste do instrumento.

Foi realizada estatística descritiva para as características demográficas, socioeconômicas e de comportamentos relacionados à saúde bucal, e avaliada a normalidade da distribuição dos dados com base no teste de Shapiro-Wilk. Os níveis de LSB foram definidos de forma semelhante ao critério sugerido pelos estudos de referência utilizados por Sistani et al.^24^, em que o nível mais baixo é expresso pelo intervalo de valores que divide a escala pela metade, sendo os demais níveis distribuídos igualmente entre os valores restantes. Dessa forma, escores entre 0 e 6 foram classificados como nível baixo/inadequado; escores de 7 a 10, nível intermediário; e 11 a 14, nível adequado/alto.

A validade convergente foi examinada comparando as diferenças dos escores médios totais e por domínios do questionário de acordo com variáveis socioeconômicas, demográficas, o tempo desde a última consulta odontológica e tipo de serviço utilizado, por meio dos testes Mann-Whitney U e Kruskal-Wallis, utilizando o programa estatístico Jamovi^®^, versão 2.3.28.

O cálculo do tamanho amostral ainda é um desafio para a modelagem por equações estruturais. Abordagens tradicionais sugerem amostras compostas, no mínimo, de 200 indivíduos, ou atender idealmente 20 observações por parâmetro. Métodos mais recentes realizam diferentes simulações^32^. Considerando que o tamanho da amostra envolveu mais de 280 observações, considerou-se que o estudo atendeu aos parâmetros de ambas as abordagens para assegurar um poder de 80% sob um alfa de 5%.

#### Declaração de Disponibilidade de Dados:

Todo o conjunto de dados que dá suporte aos resultados deste estudo está disponível mediante solicitação ao autor correspondente Moura HS. O conjunto de dados não está publicamente disponível para proteger a identidade e privacidade dos participantes e cumprir as exigências éticas.

## RESULTADOS

Doze observações com dados incompletos foram excluídas, e 348 questionários válidos foram analisados.

O instrumento com 15 itens apresentou boa consistência interna (alfa de Cronbach = 0,71) e adequação das variáveis para AFE: teste de esfericidade de Bartlett (p=0,000) e KMO = 0,77. A AFE indicou cinco fatores principais (autovalores > 1). Após rotação pelo método ortogonal Varimax, os 5 fatores explicaram 51,7% da variabilidade dos achados. No entanto, o item 11, correspondente à questão 8, “Qual é a melhor decisão se um pequeno sangramento acontecer após escovar os dentes ou usar o fio dental?”, apresentou carga < 0,4^30^, evidenciando que não avaliava nenhum dos fatores retidos – sendo, portanto, removido do instrumento. A remoção dessa questão resultou em um instrumento final com 14 itens pontuáveis distribuídos em 11 questões, e um escore que variava de 0 a 14 pontos. Assim, uma segunda AFE foi efetuada com 14 itens. A boa consistência interna (α=0,71) e a adequação para a análise fatorial (KMO = 0,77) foram mantidas.

A Tabela 1 apresenta os pesos fatoriais dos itens do questionário BOHL-YQ após AFE com rotação ortogonal, distribuídos por cinco fatores. De acordo com a natureza das questões, sete itens do questionário associados ao domínio Cognitivo (itens 1, 2, 3, 4, 5, 6 e 12) apresentaram carga fatorial mais elevada nos Fatores 2, 4 e 5, enquanto os Fatores 1 e 3 concentraram itens voltados à Compreensão e Tomada de decisão (itens 7, 8, 9, 10, 11, 13 e 14) – sugerindo a testagem de uma estrutura bidimensional. Em caso de pesos acima de 0,4 em mais de um fator, optou-se por escolher o maior peso e alocar o item no domínio que melhor se encaixava. A maioria dos itens apresentou baixa unicidade (<0,50), indicando boa comunalidade entre os itens e os fatores extraídos.

**Tabela 1:** Pesos dos fatores distribuídos por domínios segundo os itens do questionário *Brazilian Oral Health Literacy – Youngs Questionnaire* (BOHL-YQ), após rotação ortogonal (n=348).

Domínio	Pesos						
	Variável	Fator 1	Fator 2	Fator 3	Fator 4	Fator 5	Unicidade
Cognitivo	Item 1	0,11	-0,04	-0,05	0,11	**0,76**	0,39
	Item 2	0,43	**0,54**	0,00	0,11	0,11	0,50
	Item 3	0,07	**0,71**	0,00	0,16	-0,19	0,43
	Item 4	0,42	**0,58**	0,05	-0,02	-0,08	0,48
	Item 5	0,14	0,03	-0,06	**0,74**	-0,05	0,42
	Item 6	-0,02	0,08	0,09	**0,78**	0,18	0,35
	Item 12	-0,11	**0,62**	0,21	-0,01	0,39	0,41
Compreensão e tomada de decisão	Item 7	**0,52**	0,17	0,10	0,35	-0,22	0,51
	Item 8	0,09	0,08	**0,57**	0,15	0,20	0,57
	Item 9	0,18	-0,06	**0,74**	0,05	-0,02	0,42
	Item 10	**0,64**	0,13	0,29	0,10	-0,16	0,45
	Item 11	0,07	0,26	**0,62**	-0,11	-0,12	0,51
	Item 13	**0,77**	0,01	0,03	0,01	0,17	0,37
	Item 14	**0,58**	0,15	0,18	0,01	0,35	0,49

Nota: Destaque em negrito para valores acima de 0,4 que explicam cada fator.

Em seguida, a análise confirmatória para uma estrutura bidimensional foi realizada por modelagem de equação estrutural. Os valores de ajuste obtidos foram: índice da qualidade de ajuste comparativa CFI = 0,88; índice da qualidade de ajuste não normal TLI = 0,86; raiz aproximada do erro quadrático médio RMSEA = 0,05 (IC90% 0,03–0,06); resíduo do erro quadrático médio padronizado SRMR = 0,05; qui-quadrado/graus de liberdade p=0,000; coeficiente de determinação CD = 0,84, ou seja, o modelo que explicou 84% da variabilidade dos achados. O coeficiente Ω=0,72 indicou boa consistência interna geral da escala, sugerindo que os itens medem adequadamente um mesmo construto latente.

Na Figura 1 são apresentados os coeficientes ajustados para os 14 itens do questionário BOHL-YQ (Tabela Suplementar 1) segundo o modelo de equação estrutural. Quanto à distribuição da amostra de acordo com o nível de LSB, demonstrada na Figura 2, 21,3% dos adolescentes apresentaram nível baixo/inadequado; 52%, nível intermediário; e 26,7%, nível alto/adequado.

**Figura 1:** Coeficientes padronizados (valores na linha) considerando IC95%, cargas fatoriais padronizadas (valores dentro do retângulo) e variância residual estimada para os 14 itens do questionário *Brazilian Oral Health Literacy – Youngs Questionnaire* (BOHL-YQ), segundo o modelo de equação estrutural.

**Figura 2:** Distribuição da população do estudo: (A) segundo os escores de literacia em saúde bucal; e (B) de acordo com os níveis de LSB baixo/inadequado (escores de 0 a 6), intermediário (escores de 7 a 10) e adequado/alto (escores de 11 a 14).

A Tabela 2 apresenta os escores médios em cada domínio e o escore total segundo as características demográficas, socioeconômicas e de comportamentos relacionados à saúde bucal. Do total, 62,6% da amostra eram meninas, 63,8% autodeclararam-se de cor parda ou preta, 93,1% afirmaram possuir celular; 53,2% residiam em área urbana, 39,4% em casa com até 3 moradores, contando com o próprio adolescente; 61,5% afirmaram que a família recebia algum benefício do governo, 35,3% que a mãe não estudou ou não completou o ensino fundamental; 54,6% relataram ter visitado o dentista há menos de 1 ano e 60,9%, ter utilizado serviço público para a consulta.

**Tabela 2:** Escore médio e desvio-padrão total e em cada domínio do *Brazilian Oral Health Literacy – Youngs Questionnaire* (BOHL-YQ) segundo características socioeconômicas, demográficas e de comportamentos relacionados à saúde bucal da população do estudo.

Variáveis	n	%	Cognitivo	Compreensão e tomada de decisão	Escore total
			Média (DP)	Média (DP)	Média (DP)
**Sexo**					
Feminino	218	62,6	4,20 (1,39)	4,66 (1,66)^**^	8,86 (2,63)^**^
Masculino	130	37,4	3,97 (1,49)	4,12 (1,87)	8,08 (2,84)
**Cor de pele autodeclarada**					
Branco	108	31,0	4,26 (1,43)	4,67 (1,84)	8,93 (2,78)
Não branco	240	69,0	4,05 (1,43)	4,36 (1,71)	8,41 (2,70)
**Tem celular?**					
Sim	324	93,1	4,11 (1,44)	4,50 (1,75)	8,61 (2,72)
Não	24	6,9	4,13 (1,39)	3,88 (1,80)	8,00 (2,84)
**Área de residência**					
Zona urbana	185	53,2	4,31 (1,33)^**^	4,75 (1,76)^***^	9,06 (2,56)^***^
Zona rural	163	46,8	3,88 (1,51)	4,13 (1,70)	8,01 (2,82)
**Quantidade de moradores**					
Até 3	137	39,4	4,26 (1,49)	4,53 (1,75)	8,80 (2,81)
4	109	31,3	4,07 (1,43)	4,61 (1,76)	8,69 (2,76)
5 ou mais	102	29,3	3,95 (1,34)	4,19 (1,74)	8,14 (2,58)
**Escolaridade da mãe**					
≤9 anos	196	57,5	4,03 (1,44)	4,38 (1,80)	8,40 (2,83)
>9 anos	122	35,8	4,24 (1,36)	4,72 (1,64)^**^	8,96 (2,48)^**^
Não sabe	23	6,7	3,74 (1,57)	3,57 (1,56)	7,30 (2,62)
**Benefício governamental**					
Sim	214	61,5	3,97 (1,49)	4,36 (1,75)	8,32 (2,79)
Não	93	26,7	4,56 (1,28)^***^	4,91 (1,63)^**^	9,47 (2,40)^***^
Não sabe	41	11,8	3,85 (1,26)	3,95 (1,90)	7,80 (2,71)
**Última consulta no dentist**a					
Até 1 ano	190	54,6	4,27 (1,41)	4,80 (1,71)^***^	9,07 (2,68)^***^
Mais de 1 ano	116	33,3	3,98 (1,46)	4,03 (1,76)	8,02 (2,73)
Não sabe	42	12,1	3,71 (1,35)	4,00 (1,63)	7,71 (2,44)
**Local da última consulta**					
Serviço público	212	60,9	4,09 (1,44)	4,42 (1,74)	8,51 (2,73)
Serviço privado	102	29,3	4,27 (1,37)	4,72 (1,78)	8,99 (2,60)^*^
Não sabe	34	9,8	3,74 (1,54)	3,91 (1,71)	7,65 (2,92)^*^
**Total**	**348**	**100**			

DP: desvio-padrão;

*p<0,05;

**p≤0,01;

***p≤0,001.

Quanto à validade convergente, escores médios de LSB estatisticamente mais elevados foram observados entre adolescentes do sexo feminino (p=0,006), que residiam na área urbana (p<0,001), cujas mães tinham mais de 9 anos de estudo (p=0,014), que não recebiam benefício do governo (p<0,001), que realizaram a última consulta odontológica há até um ano (p<0,001) e utilizaram o serviço privado para a consulta (p=0,043). Os escores médios foram mais favoráveis para o domínio Cognitivo entre residentes da área urbana (p=0,003), que não participavam de programa de benefício do governo (p<0,001) e com última consulta no dentista há até um ano (p=0,023); enquanto para o domínio Compreensão e Tomada de decisão foram identificadas maiores médias para o sexo feminino (p=0,005), escolaridade materna superior a 9 anos (p=0,011), os que residiam em área urbana (p=0,001), não recebiam benefício do governo (p=0,013) e tiveram última consulta odontológica há até um ano (p<0,001).

## DISCUSSÃO

O instrumento BOHL-YQ testado para mensurar a literacia em saúde bucal de adolescentes brasileiros de 15 a 19 anos apresentou adequada consistência interna, boa validade estrutural e validade convergente. Os resultados foram semelhantes aos da versão iraniana original (α=0,72)^24^ e da versão brasileira (α=0,73)^22^, ambas voltadas para adultos.

O BREALD-30, utilizado frequentemente em adolescentes brasileiros, identificou consistência interna maior que o BOHL-YQ (α=0,83), mas resultados de validade convergente semelhantes: maior nível de LSB entre adolescentes de melhores condições econômicas e pais de maior escolaridade^7^.

O escore médio da LSB foi maior entre adolescentes do sexo feminino, resultado compatível ao encontrado por Lima et al.^7^ em adolescentes de 12 anos. A relação entre maior escore de LSB, maior escolaridade materna e melhor situação econômica familiar, representada neste estudo pela ausência de qualquer vínculo a programa assistencial do governo, também foi observada por Lopes et al.^5^ com adolescentes de mesma faixa etária.

Adolescentes que relataram ter realizado a última consulta odontológica há até um ano apresentaram maiores médias de LSB, no escore total e por domínios. Valores mais elevados de LSB parecem estar relacionados a maior consciência de cuidado com a saúde bucal na adolescência e, assim, a comportamentos mais positivos.

Ainda que a distribuição das características da população do estudo tenha permitido a detecção de diferenças entre os valores de LSB e as variáveis demográficas, socioe-conômicas e de comportamentos relacionados à saúde bucal, como a validação de instrumentos envolve síntese de evidência de diferentes fontes, sugere-se que este instrumento seja testado em diferentes contextos socioeconômicos e culturais brasileiros. Do total de adolescentes, 21,3% apresentaram nível baixo/inadequado de LSB. Como a distribuição da escala de valores de LSB em três níveis acompanhou sugestão da literatura pregressa^33,34^, recomenda-se que a classificação proposta seja objeto de escrutínio por futuros estudos.

Embora não tenham sido investigados todos os parâmetros para análise psicométrica de instrumentos de aferição de desfechos relacionados à saúde^24^, o BOHL-YQ apresentou resultados satisfatórios em todas as análises propostas. A AFE indicou uma estrutura de cinco fatores, com distribuição coerente entre os domínios teóricos propostos: Cognitivo e Compreensão/Tomada de decisão. A opção pela rotação ortogonal representou uma abordagem compatível com o caráter exploratório da análise, favorecendo uma solução mais parcimoniosa e de interpretação mais direta dos agrupamentos fatoriais. Entretanto, considerando tratar-se de um construto psicossocial multidimensional, não se pode excluir a possibilidade de correlação entre os domínios identificados, e estudos futuros poderão explorar rotações oblíquas como estratégia complementar para avaliar a estabilidade e a interpretabilidade da estrutura encontrada.

Na AFC, os índices RMSEA e SRMR apresentaram valores nos limites adequados, enquanto os índices TLI (0,86) e CFI (0,88) ficaram no limite do parâmetro de referência descrito na metodologia. Como na avaliação dos resultados deve-se empregar um conjunto de medidas ao invés de apenas um valor de referência^35^, o ajuste do modelo foi considerado satisfatório e teoricamente coerente com a estrutura proposta. Assim, optou-se por manter o modelo, reconhecendo a limitação e destacando que os índices de qualidade de ajuste constituem apenas um aspecto da avaliação do modelo^35^.

Embora informado pelos autores que o questionário original utilizado como base para o instrumento validado neste estudo avaliasse quatro dimensões relacionadas a componentes conceituais da LSB (compreensão de leitura, numeracia, escuta e tomada de decisão)^22^, foi reportada apenas a AFC unidimensional do instrumento.

Os valores satisfatórios de consistência interna e a AFC demonstram que a estrutura final do instrumento apresentou boas propriedades psicométricas, mantendo a coerência teórica e melhorando a interpretabilidade dos resultados. A fusão em dois domínios mais abrangentes (cognitivo e compreensão/tomada de decisão) resultou em uma estrutura mais parcimoniosa. A avaliação desses dois domínios do construto LSB por um único instrumento permite uma análise mais detalhada e pode indicar pontos fortes e fracos para cada um deles, auxiliando o delineamento e monitoramento de possíveis intervenções práticas de LSB com adolescentes.

No presente estudo, dois itens apresentaram carga fatorial adequada em mais de um fator, o que pode estar relacionado às características da população do estudo. Em que pese a estrutura geral indicar agrupamento teórico satisfatório, a sobreposição de um item em mais de um fator sugere a necessidade de refinamento do item por meio de revisões futuras na escala, na redação de alguns itens e na possibilidade de testagem com rotação oblíqua, que pode capturar correlações entre os fatores. Contudo, a AFE e a AFC foram realizadas na mesma amostra, o que limita a robustez da evidência de validade estrutural. Embora essa estratégia possa ser útil em avaliações psicométricas iniciais, a ausência de divisão amostral, validação cruzada ou teste em amostra independente pode produzir estimativas de ajuste mais otimistas. Assim, os achados devem ser interpretados como evidência preliminar da estrutura fatorial do BOHL-YQ, a ser aprofundada em investigações futuras com replicação em outras populações adolescentes.

Também pode ser considerada uma limitação o fato de não ter sido avaliada a validade convergente comparando o BOHL-YQ a outro instrumento de LSB previamente validado para a mesma população. Ainda assim, os resultados sugerem adequação psicométrica preliminar do instrumento para aplicação em populações adolescentes brasileiras, justificando sua validação confirmatória em estudos subsequentes.

Comparado ao BREALD-30, o qual necessita de entrevistador e mede o reconhecimento de termos^17^, o BOHL-YQ pode apresentar vantagens ao considerar a complexidade e multidimensionalidade da literacia. Para além de considerar o conhecimento prévio do indivíduo sobre saúde bucal, ao apresentar etapas de interpretação, cálculo e decisões diante de problemas básicos relacionados à saúde bucal, o BOHL-YQ tem capacidade de avaliar do aspecto cognitivo à compreensão e tomada de decisão. Ademais, esta versão autoaplicável do questionário adiciona a praticidade da possibilidade de testagem e do uso deste instrumento em diferentes contextos.

O HeLD-14 mensura a LSB utilizando questões baseadas na autopercepção do indivíduo sobre seu grau de dificuldade para desempenhar atividades relacionadas à saúde bucal^20^. Apesar do BOHL-YQ medir apenas duas dimensões, pode significar uma vantagem para a interpretação do escore global de LSB comparado ao HeLD-14, cujo escore é resultante de sete domínios, alguns dos quais bem distintos^20^.

No contexto investigado, concluiu-se que o BOHL-YQ apresentou boa consistência interna e validade convergente, demonstrando ser um instrumento válido e de fácil aplicação para mensurar a LSB entre adolescentes brasileiros.

## MATERIAL SUPLEMENTAR

Tabela Suplementar 1

## COMITÊ DE ÉTICA:

Este trabalho foi aprovado pelo Comitê de Ética em Pesquisa da Faculdade de Saúde Pública da Universidade de São Paulo (FSP/USP), nº do Parecer 6.316.539/CAAE 73709623.1.0000.5421.
